# A meta-analysis of randomized controlled trials examining the effectiveness of carbetocin in reducing intraoperative blood loss during abdominal myomectomy

**DOI:** 10.3389/fmed.2025.1590144

**Published:** 2025-06-11

**Authors:** Ahmed Abu-Zaid, Saeed Baradwan, Bandr Hafedh, Majed Saeed Alshahrani, Maha Al Baalharith, Ehab Badghish, Fahad Algreisi, Mohammed Ziad Jamjoom, Saad M. S. Alqarni, Hedaya Albelwi, Ahmed Nazer, Afnan Baradwan, Saud Owaimer Alsehaimi, Mohannad Alsabban, Osama Alomar

**Affiliations:** ^1^College of Medicine, Alfaisal University, Riyadh, Saudi Arabia; ^2^Department of Obstetrics and Gynecology, King Faisal Specialist Hospital and Research Center, Jeddah, Saudi Arabia; ^3^Department of Obstetrics and Gynecology, Faculty of Medicine, Najran University, Najran, Saudi Arabia; ^4^Department of Obstetrics and Gynecology, King Abdulaziz Medical City, Riyadh, Saudi Arabia; ^5^Department of Obstetrics and Gynecology, Maternity and Children Hospital, Makkah, Saudi Arabia; ^6^Department of Obstetrics and Gynecology, Jeddah University, Jeddah, Saudi Arabia; ^7^Department of Obstetrics and Gynecology, King Fahad Armed Forces Hospital, Jeddah, Saudi Arabia; ^8^Department of Obstetrics and Gynecology, King Faisal Armed Forces Hospital, Khamis Mushait, Saudi Arabia; ^9^Department of Obstetrics and Gynecology, College of Medicine, Tabuk University, Tabuk, Saudi Arabia; ^10^Department of Obstetrics and Gynecology, King Faisal Specialist Hospital and Research Center, Riyadh, Saudi Arabia; ^11^Department of Obstetrics and Gynecology, Al Salama Hospital, Jeddah, Saudi Arabia

**Keywords:** myomectomy, leiomyoma, bleeding, meta-analysis, carbetocin

## Abstract

**Aim:**

This study aimed to systematically review and meta-analyze randomized controlled trials (RCTs) assessing the clinical efficacy and safety of carbetocin compared to passive control (placebo or no treatment) in the context of abdominal myomectomy.

**Methods:**

Six sources of information underwent screening until 13 April 2024. The risk of bias was assessed using the Cochrane Collaboration tool. The results were presented as mean difference (MD) or risk ratio (RR) along with a 95% confidence interval (CI) using a random-effects model.

**Results:**

Five RCTs with 6 arms and 484 patients (carbetocin = 262 and control = 222) were analyzed. The overall risk of bias was “low” in two studies and “some concerns” in three studies. The carbetocin group exhibited significantly lower mean intraoperative blood loss (*n* = 6 arms, MD = –292.27 mL, 95% CI [−372.5, −212.03], *p* < 0.001, with very low certainty of evidence), mean change in hemoglobin (*n* = 6 arms, MD = –0.63 g/dL, 95% CI [−0.94, −0.33], *p* < 0.001, with low certainty of evidence), rate of blood transfusion (RR = 0.3, 95% CI [0.21, 0.44], *p* < 0.001, with very low certainty of evidence), and mean operation time (*n* = 5 arms, MD = −22.98 min, 95% CI [−38.93, −7.02], *p* < 0.001, with low certainty of evidence). There was no significant difference between both groups regarding the mean hospital stay (*n* = 2 arms, MD = –0.1 days, 95% CI [−0.27, 0.06], *p* = 0.21). The sensitivity analyses demonstrated robustness across all outcomes. No major toxicities were reported.

**Conclusion:**

Carbetocin use was tolerable and associated with considerable declines in intraoperative blood loss and related complications compared with passive control intervention during abdominal myomectomy.

## Introduction

Uterine leiomyomas are the most prevalent non-cancerous neoplasms within the female genital system worldwide. Although they often manifest without symptoms, approximately one-third of patients experience symptomatic presentations, which can lead to a diverse range of significant complications. These complications may include pelvic discomfort, heightened menstrual bleeding, and complications during pregnancy (1, 2).

Current treatment options for symptomatic uterine fibroids encompass various approaches: expectant management, pharmacological interventions, surgical procedures, and interventional radiology techniques. Surgery becomes the primary consideration when expectant and pharmacological methods prove ineffective in symptom management (1, 2).

Uterine leiomyomas, being highly vascular tumors (3), pose a significant risk of bleeding during myomectomy (4). Additionally, myomectomy itself carries inherent risks, with perioperative blood loss being a commonly reported complication (5). This loss of blood can escalate to the point where urgent blood transfusions are necessary. Furthermore, severe hemorrhage during myomectomy can lead to life-threatening complications such as hemodynamic instability, shock, coagulopathy, and even death, if not managed effectively (5). Therefore, implementing strategies to reduce bleeding and its associated morbidities during myomectomy is imperative for reducing both morbidity and mortality.

An increasing body of research has investigated the effectiveness of numerous perioperative pharmacological methods aimed at reducing bleeding and its associated complications during myomectomy. In this context, carbetocin has gained recognition for its use in preventing postpartum hemorrhage (6, 7). Carbetocin, a synthetic octapeptide with prolonged action, is structurally close to oxytocin and acts as an agonist for oxytocin receptors. It binds to oxytocin receptors in uterine smooth muscles, thereby inducing recurring contractions (8, 9).

The clinical benefits of perioperative carbetocin administration during myomectomy remain inadequately defined. To date, several randomized controlled trials (RCTs) have investigated the benefit of perioperative carbetocin compared to passive control in patients undergoing myomectomy (10–14). However, these studies have yielded inconsistent results and have been constrained by small sample sizes. Moreover, no study has systematically reviewed the existing literature to date. To address this gap, we conducted this first-ever meta-analysis of RCTs aiming to comprehensively gather robust evidence regarding the clinical efficacy and tolerability of perioperative carbetocin compared with passive control during abdominal myomectomy.

## Methods

We conducted this study following the guidelines outlined in the Cochrane Handbook for Systematic Reviews of Interventions (15) and the Preferred Reporting Items for Systematic Reviews and Meta-Analyses (PRISMA) statement (16). Ethical approval was not required as the study did not involve any individual patient data.

We included studies that met the following conditions: (i) patients underwent abdominal myomectomy, (ii) the experimental group received perioperative carbetocin, (iii) the control group received either a placebo or no treatment, (iv) the primary endpoint of intraoperative blood loss was reliably reported, and (v) the study design was RCTs. We omitted studies that failed to meet these conditions, including non-randomized studies, studies using drugs other than carbetocin, and studies comparing carbetocin with an active intervention (e.g., uterine artery tourniquet).

We conducted searches across six databases: Cochrane Central Register of Controlled Trials, Embase, PubMed, Scopus, Google Scholar, and Web of Science. The exact search strategy utilized in the databases is outlined in Supplementary Table 1 and comprised the following query: (carbetocin OR duratocin OR papal) AND (myomectomy). No filters were applied during the search process, including language, publication date, or geography. Database screening encompassed records from inception until 13 April 2024. Following the removal of duplicates, we screened abstracts and titles, excluding irrelevant citations. Subsequently, a full-text review determined the final inclusion of appropriate studies in our analysis. To curtail the risk of overlooking relevant studies, we inspected the reference lists of included RCTs and the latest reviews. Two coauthors conducted the database search independently, resolving disagreements through consensus.

We gathered data on author names, date of publication, country, sample sizes, participant ages, participant body mass indices, number of leiomyomas, dimension of the largest leiomyoma, type of uterine fibroids, and details of perioperative carbetocin and control interventions. The average intraoperative blood loss was the primary endpoint. The average change in hemoglobin levels (postoperative minus preoperative), rate of perioperative blood transfusion (defined as transfusions administered from the intraoperative period through the postoperative period until discharge), average operative time, average hospital stay, and safety profile were the secondary endpoints. Two groups, each consisting of two coauthors, independently extracted the information, resolving any discrepancies through consensus within each group.

We evaluated the quality of the included RCTs using the Cochrane risk of bias assessment tool, version 2 (17). We also appraised the quality of evidence according to the Grading of Recommendations, Assessment, Development, and Evaluation (GRADE) approach (18). Two coauthors conducted the judgments independently, resolving any disagreements through mutual agreement.

Notably, one study (13) included two arms (intravenous and intramyometrial administration of carbetocin), each regarded as a single RCT during meta-analysis. The intravenous RCTs were labeled as Sayed 2022 IV, while the intramyometrial RCTs were labeled as Sayed 2019 IM. Using the random-effects model (19), we summarized data as mean difference (MD) and risk ratio (RR), accompanied by 95% confidence interval (CI). Assessment of between-study heterogeneity was conducted based on Higgins’ I^2^ statistic >50% (20). Additionally, leave-one-out sensitivity analyses were completed to evaluate the strength of conclusions, whereby one RCT was sequentially excluded to recalibrate summary effect sizes for the remaining RCTs. Subgroup analysis based on the route of drug administration (intravenous vs. intramyometrial) was conducted for heterogeneous outcomes to explore potential sources of heterogeneity. Publication bias is examined both qualitatively using funnel plots and quantitatively using Egger’s regression test. STATA software was used for data analysis. Statistical significance was specified as a *p*-value of <0.05 for all endpoints.

## Results

**Figure 1:**
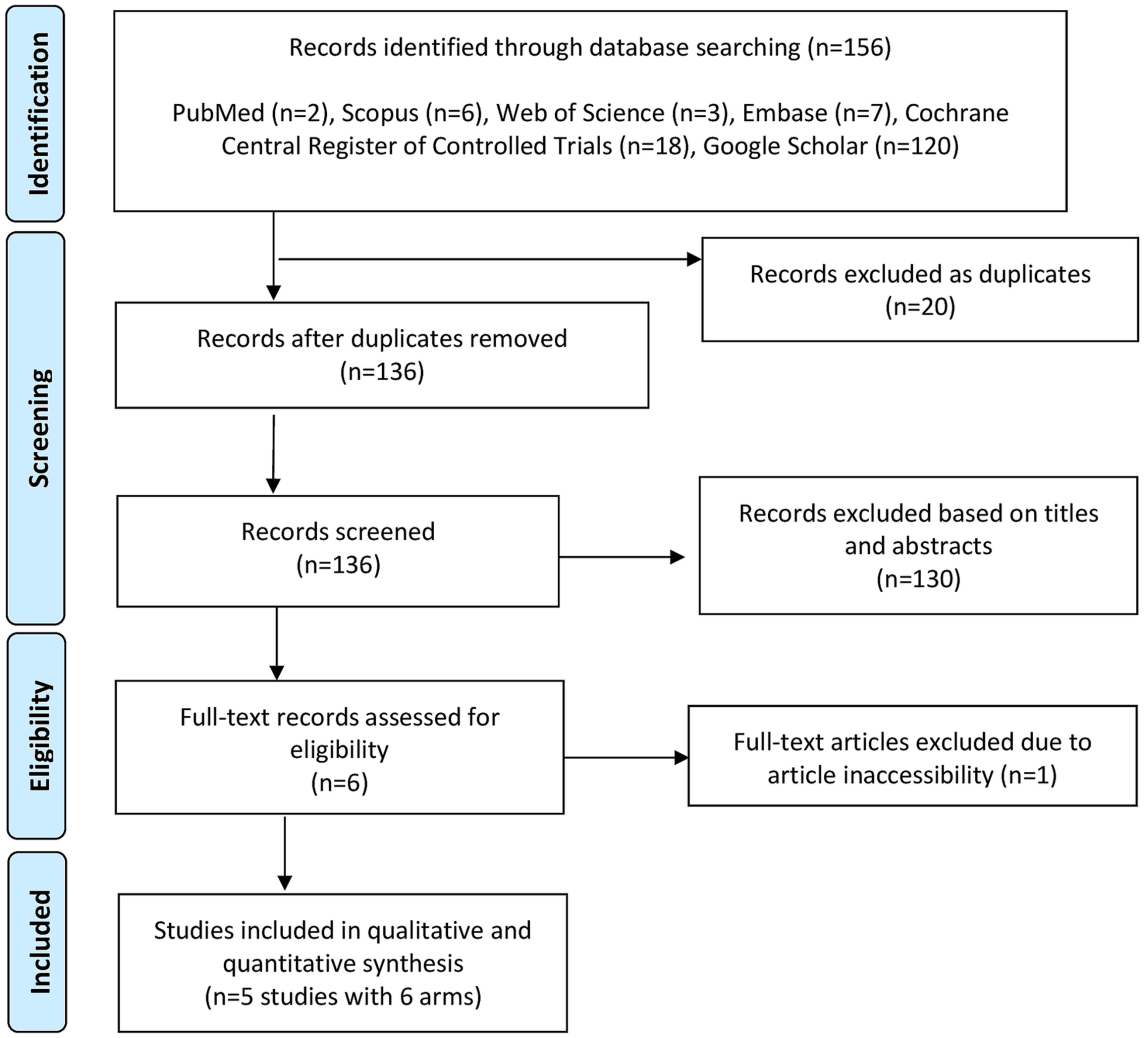
PRISMA flowchart for literature search and study selection.

Figure 1 shows the PRISMA flowchart for database screening. Overall, five studies with 6 arms and 484 patients (carbetocin = 262 and control = 222) were included in the analysis (10–14).

Table 1 shows the summary of the included RCTs. All studies were conducted in Egypt from 2017 to 2023 and utilized the abdominal approach for myomectomy. Carbetocin (100 μg) was administered intravenously in four arms and intramyometrially in two arms. The type of control intervention was no treatment and a matched placebo in four and two arms, respectively. There were no substantial distinctions between the two groups in terms of age, number of leiomyomas, and dimension of the largest leiomyoma.

**Table 1:** Baseline characteristics of the included studies.

Study identifier	Study arms	*n*	Intervention	Age (year)	Body mass index (kg/m^2^)	Number of leiomyomas	Size of largest leiomyoma (cm)	Types of leiomyoma
Sallam 2017 (10)	Carbetocin	43	Intravenous (100 μg)	34.84 ± 4.41	25.58 ± 2	3 (1–5)	13.21 ± 3.41	NR
	Control	43	Matched placebo	34.49 ± 4.39	25.43 ± 2.06	3 (1–5)	13.09 ± 3.85	
Mohamed 2021 (12)	Carbetocin	40	Intramyometrial (100 μg)	45.67 ± 3.85	25.2 ± 1.86	1.5 ± 0.5 (1–3)	8.08 ± 1.2 (6–10)	Intramural, submucosal, subserosal
	Control	40	Nothing	44.52 ± 3.85	26.5 ± 1.85	1.5 ± 0.05 (1–3)	7.5 ± 1.1 (5–10)	
Taher 2021 (11)	Carbetocin	69	Intravenous (100 μg)	44.4 ± 3.2	26.7 ± 3.6	2.7 ± 1.2	14.51 ± 12.6	NR
	Control	69	Matched placebo	44.01 ± 3.4	26.5 ± 3.9	2.9 ± 1.1	14.61 ± 12.8	
Sayed 2022 IM (13)	Carbetocin	40	Intramyometrial (100 μg)	34.05 ± 3.72	68.35 ± 5.18	NR	6.45 ± 1.11	NR
	Control	40	Nothing	33.2 ± 4.69	69.30 ± 5.25	NR	5.98 ± 0.85	
Sayed 2022 IV (13)	Carbetocin	40	Intravenous (100 μg)	34.70 ± 4.28	74.20 ± 5.71	NR	7.20 ± 1.38	NR
	Control	40	Nothing	33.2 ± 4.69	69.30 ± 5.25	NR	5.98 ± 0.85	
Mousad 2023 (14)	Carbetocin	30	Intravenous (100 μg)	37.10 ± 4.35	69.97 ± 9.69	NR	10.67 ± 3.21	Intramural
	Control	30	Nothing	36.67 ± 3.70	66.90 ± 6.46	NR	10.45 ± 3.06	

Age and body mass index were reported as mean ± standard deviation (minimum–maximum) or median (minimum–maximum).

**Figure 2:**
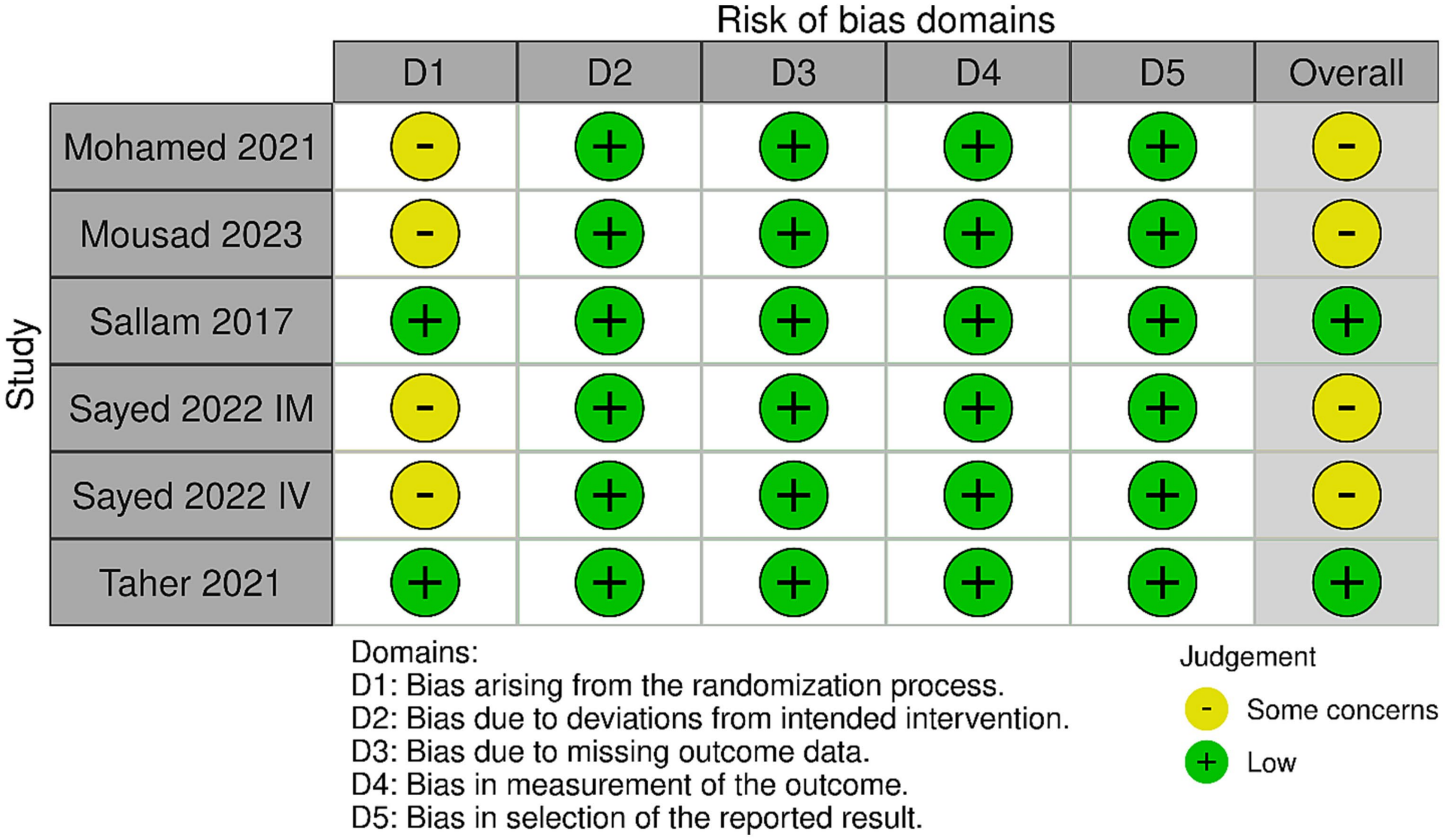
Summary of the risk of bias of the included studies.

Figure 2 shows the results of the risk of bias. Two studies had an overall “low” risk of bias (10, 11) and three studies (one study with two arms) had an overall “some concerns” risk of bias (12–14). This is because these studies did not offer sufficient description on allocation concealment; hence, the randomization process was judged as having “some concerns.”

**Figure 3:**
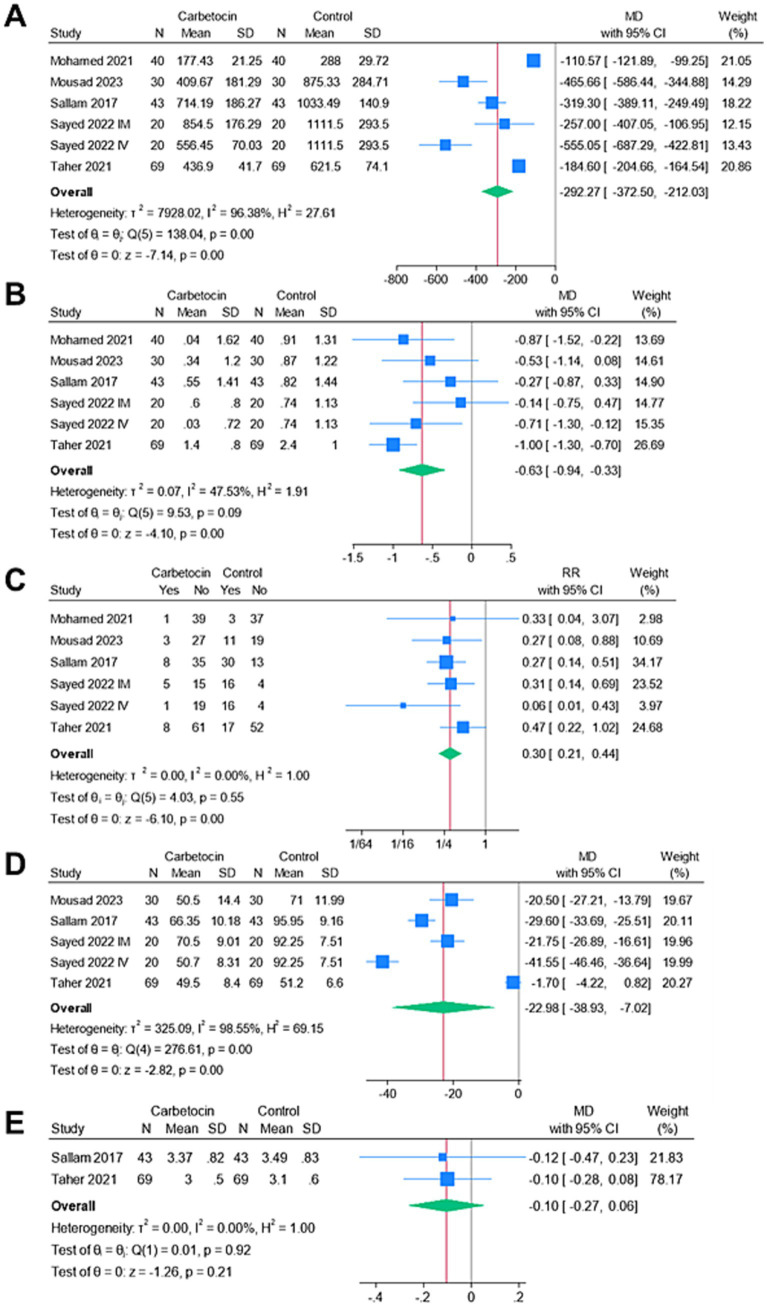
Meta-analysis of the endpoints: **(A)** mean intraoperative blood loss (ml), **(B)** mean change in hemoglobin (g/dl), **(C)** rate of blood transfusion (%), **(D)** mean operative time (mins), and **(E)** mean length of hospital stay (days).

Figure 3 shows the forest plots of the meta-analyzed endpoints. The carbetocin group exhibited significantly lower mean intraoperative blood loss (*n* = 6 arms, MD = −292.27 mL, 95% CI [−372.5, −212.03], *p* < 0.001), mean drop in hemoglobin (*n* = 6 arms, MD = −0.63 g/dL, 95% CI [−0.94, −0.33], *p* < 0.001), rate of perioperative blood transfusion (RR = 0.3, 95% CI [0.21, 0.44], *p* < 0.001), and mean operation time (*n* = 5 arms, MD = −22.98 min, 95% CI [−38.93, −7.02], *p* < 0.001). The analyses were heterogeneous for the endpoints of the mean intraoperative blood loss (I^2^ = 96.38%) and mean operation time (I^2^ = 98.55%). Conversely, the analyses were homogenous for the endpoints of the mean change in hemoglobin (I^2^ = 47.53%) and the rate of perioperative blood transfusion (I^2^ = 0%). There was no significant change between both groups for the mean duration of hospitalization (*n* = 2 arms, MD = –0.1 days, 95% CI [−0.27, 0.06], *p* = 0.21), and the analysis was homogenous (I^2^ = 0%).

**Figure 4:**
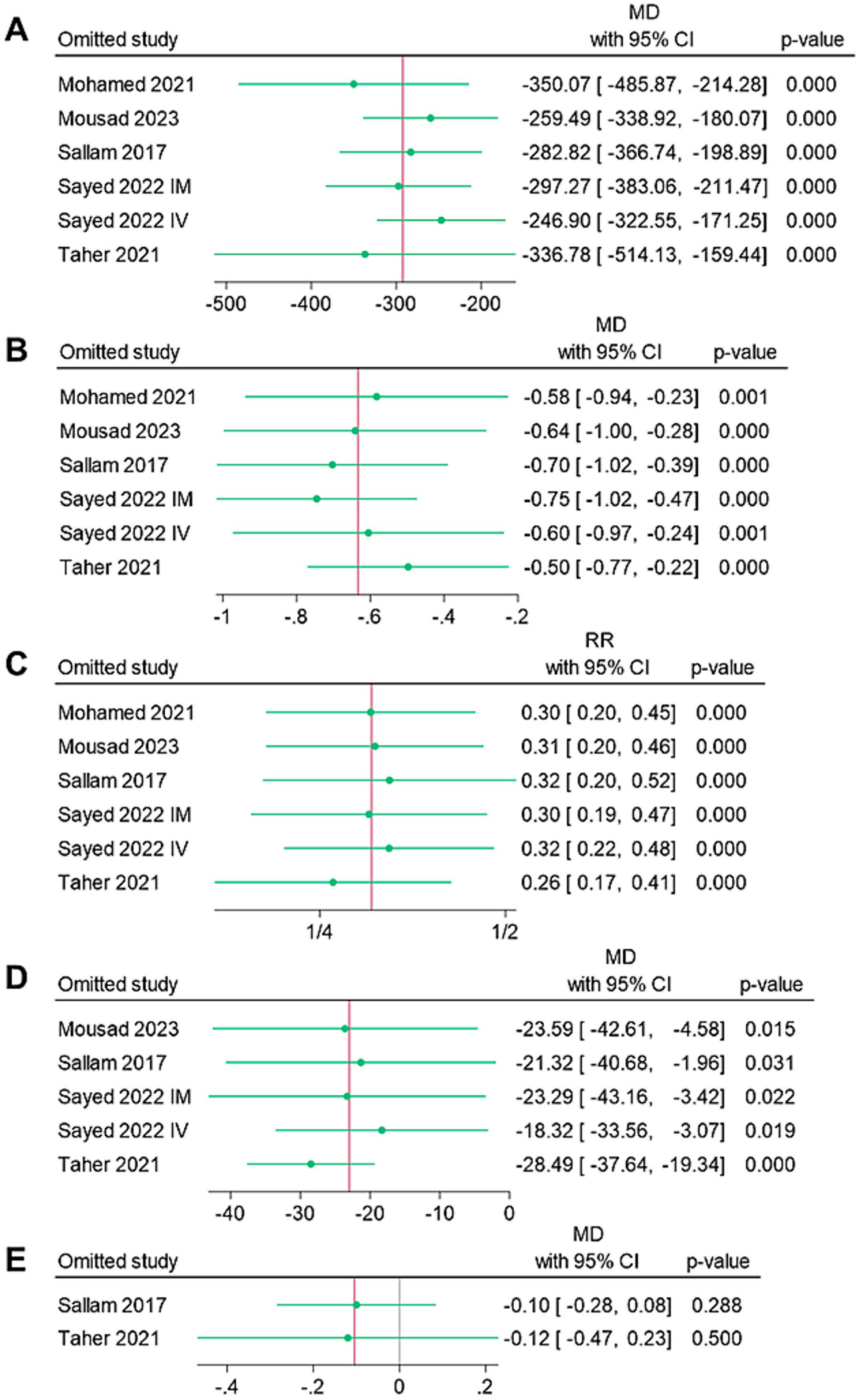
Leave-one-out sensitivity analysis of the endpoints: **(A)** mean intraoperative blood loss (ml), **(B)** mean change in hemoglobin (g/dl), **(C)** rate of blood transfusion (%), **(D)** mean operative time (mins), and **(E)** mean length of hospital stay (days).

Figure 4 shows the findings of the sensitivity analyses. All endpoints demonstrated the stability of the findings, as the omission of individual studies was not linked to significant alteration of the MD or RR summaries.

**Figure 5:**
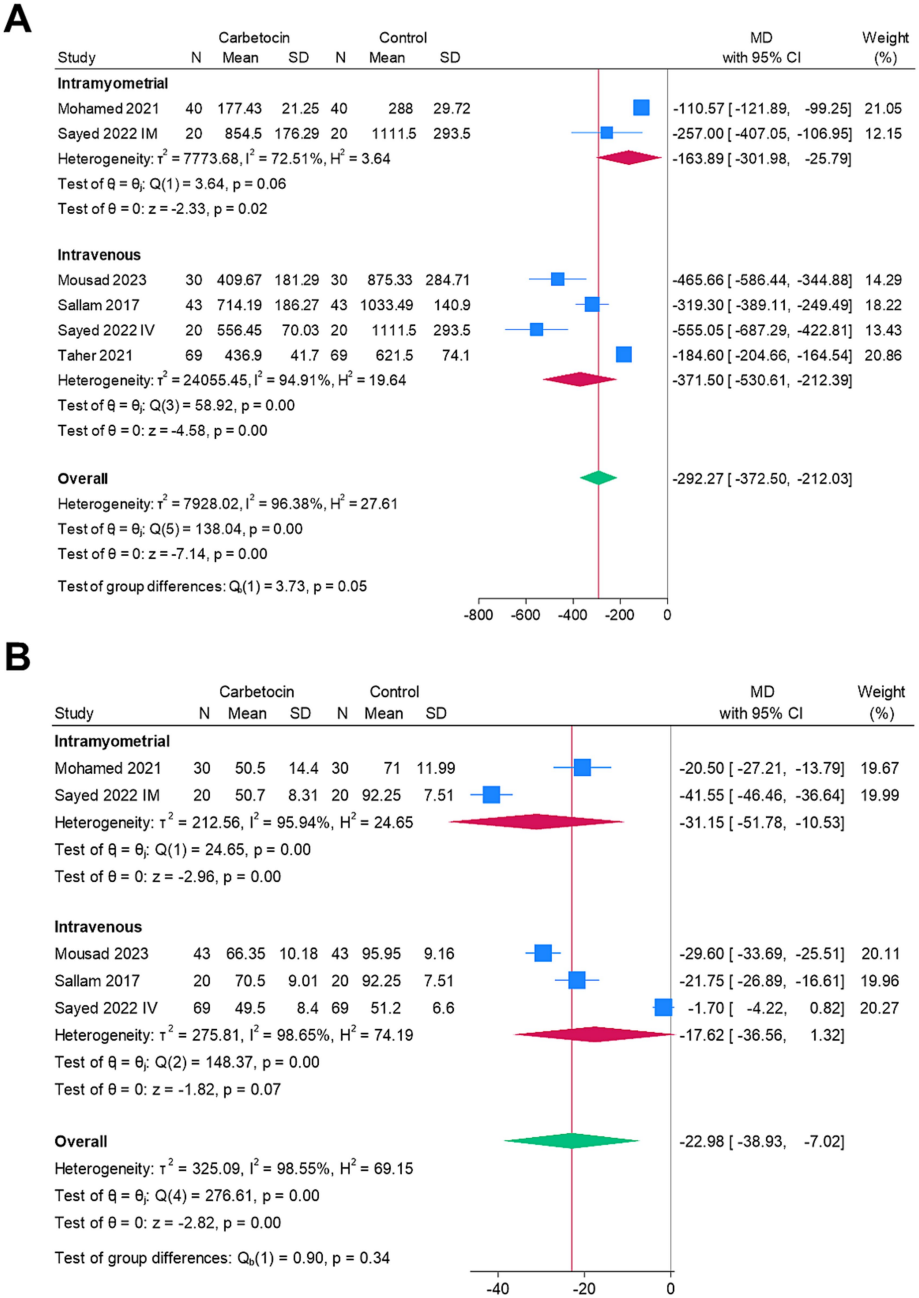
Subgroup analysis based on the route of drug administration (intravenous vs. intramyometrial) for the heterogeneous endpoints: **(A)** mean intraoperative blood loss (ml) and **(B)** mean operative time (mins).

Figure 5 shows a subgroup analysis based on the route of drug administration (intravenous vs. intramyometrial) to explore potential sources of heterogeneity in the outcomes. The carbetocin group demonstrated significantly lower mean intraoperative blood loss for both the intramyometrial (MD = –163.89 mL, 95% CI [−301.98, −25.79], *p* = 0.02) and intravenous (MD = –371.5 mL, 95% CI [−530.61, −212.39], *p* < 0.001) routes, although heterogeneity remained unresolved. Additionally, the carbetocin group had a significantly shorter mean operative time for the intramyometrial route (MD = –31.15, 95% CI [−51.78, −10.53], *p* < 0.001), but not for the intravenous route (MD = –17.62, 95% CI [−36.56, 1.32], *p* = 0.07), again with persistent heterogeneity.

**Figure 6:**
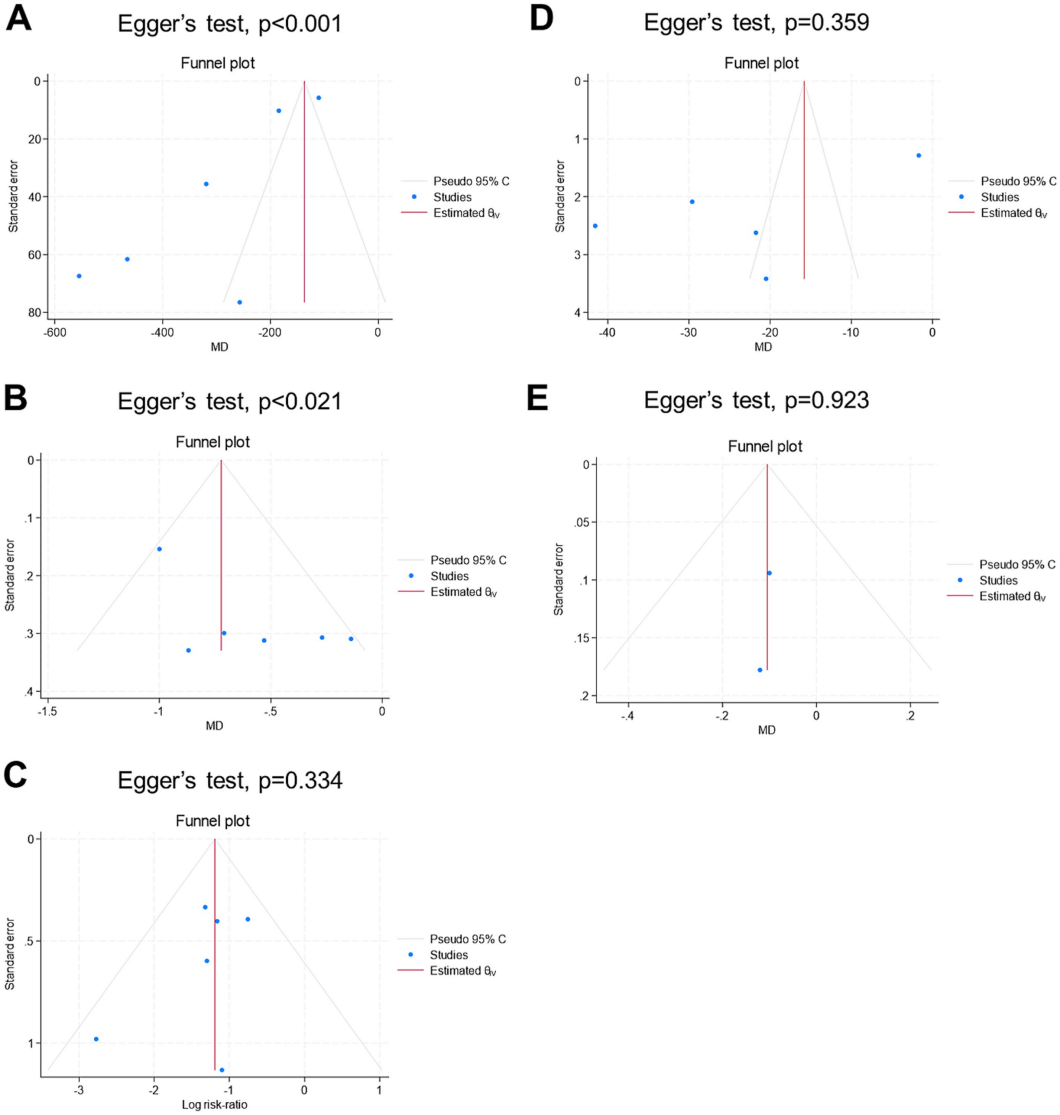
Funnel plot for publication bias assessment of the endpoints: **(A)** mean intraoperative blood loss (ml), **(B)** mean change in hemoglobin (g/dl), **(C)** rate of blood transfusion (%), **(D)** mean operative time (mins), and **(E)** mean length of hospital stay (days).

Figure 6 shows the findings related to publication bias. Visual inspection of the funnel plots revealed potential asymmetry for all endpoints, except for the mean length of hospital stay. Given the small number of studies per endpoint, the results from the funnel plots and Egger’s regression tests should be interpreted with caution.

Table 2 shows the summary of the certainty of evidence according to the GRADE method. The certainty of evidence was “very low” for the endpoints of intraoperative blood loss and perioperative blood transfusion. On the other hand, the certainty of evidence was “low” for the endpoints of change in hemoglobin, hospital stay, and operative time.

**Table 2:** Summary of the quality of evidence according to the GRADE approach.

Endpoint	Certainty assessment							Overall certainty
	# of studies	Study design	Risk of bias*	Inconsistency**	Indirectness	Imprecision***	Publication bias	
Intraoperative blood loss	6	Randomized trials	Serious	Serious	Not serious	Serious	Undetected	⨁◯◯◯ Very low
Change in hemoglobin	6	Randomized trials	Serious	Not serious	Not serious	Serious	Undetected	⨁⨁◯◯ Low
Blood transfusion	6	Randomized trials	Serious	Serious	Not serious	Serious	Undetected	⨁◯◯◯ Very low
Operative time	5	Randomized trials	Serious	Not serious	Not serious	Serious	Undetected	⨁⨁◯◯ Low
Hospital stay	2	Randomized trials	Serious	Not serious	Not serious	Serious	Undetected	⨁⨁◯◯ Low

* Risk of bias was judged as serious because most studies had some concerns regarding the allocation concealment. ** Inconsistency was judged as serious when the between-study heterogeneity was significant (I^2^ statistic>50%). *** Imprecision was judged as serious because of the small sample size.

## Discussion

We conducted this inaugural meta-analysis, encompassing five RCTs with a total of six arms, comparing perioperative carbetocin (*n* = 262 patients) against passive control (*n* = 222 patients) in the context of abdominal myomectomy. The overall risk of bias was “low” and “some concerns” in two and three studies, respectively. Compared with the passive control group, the use of carbetocin appeared to be linked to significant decreases in intraoperative bleeding, postoperative hemoglobin drop, perioperative blood transfusion, and surgery duration. These results were found to be stable during sensitivity analyses. The certainty of evidence ranged from “very low” to “low” according to the GRADE approach. Publication bias was not assessed.

Uterine leiomyomas are hypoxic neoplasms (21) with an increased tendency to have enhanced angiogenesis (3). Consequently, the heightened vascularity of uterine leiomyomas may significantly contribute to the elevated risk of intraoperative bleeding. Effective management of bleeding during this procedure is crucial, as substantial intraoperative hemorrhage can lead to various adverse outcomes, including compromised visibility in the operative vicinity, heightened jeopardy of iatrogenic damages, and an increased possibility of developing serious complications such as hemodynamic instability, the need for blood transfusions, and infections delaying wound healing (22). Therefore, women undergoing myomectomy may gain advantage from the prophylactic, perioperative intake of antihemorrhagic agents such as carbetocin, which could help mitigate these risks.

In our analysis, the use of carbetocin was associated with a mean reduction in hemoglobin levels of approximately 0.63 g/dL, which was statistically significant. A hemoglobin drop of 0.63 g/dL, while seemingly small, is clinically meaningful in the context of myomectomy, as patients often present with low hemoglobin levels due to the hemorrhagic nature of the tumors. Furthermore, given the blood loss associated with the surgery, any reduction in hemoglobin levels, even modest, can have a significant impact on patient outcomes and recovery. Therefore, this reduction in hemoglobin should be considered a relevant clinical benefit in this population.

During surgical procedures, including myomectomy, the use of pharmacological agents to enhance hemostasis, either through systemic or topical administration, is justified (23, 24). Examples of such agents include uterotonics (e.g., misoprostol and oxytocin) (25, 26), vasoconstrictors (e.g., vasopressin) (27), and antifibrinolytics (e.g., tranexamic acid) (28). All these agents exhibited efficacy in reducing intraoperative bleeding and related morbidities compared to passive (placebo or no intervention) controls. Samy et al. conducted a network meta-analysis of 17 RCTs involving 1,153 patients to evaluate various perioperative non-hormonal pharmacological interventions for reducing bleeding during abdominal myomectomy (28). The analysis found that vasopressin plus misoprostol, oxytocin, tranexamic acid, and misoprostol were effective in reducing bleeding, although the quality of evidence was low. Among these interventions, vasopressin plus misoprostol ranked first in minimizing blood loss during the procedure. At present, no single hemostatic agent can be identified as the standard of care. Apart from these agents, carbetocin has recently gained popularity as a hemostatic agent. However, the evidence regarding the use of carbetocin in myomectomy is limited and lacks a comprehensive review (10–14).

From a pharmacological perspective, carbetocin, a synthetic octapeptide, shares structural similarities with oxytocin, a nonapeptide, and exhibits agonistic effects at oxytocin receptors. By binding to oxytocin receptors in a selective fashion in uterine smooth muscles, carbetocin induces recurring contractions and promotes increased uterine tone (8). The most common dose of carbetocin is 100 μg, commonly through intravenous or intramuscular routes (9) and less commonly via the intramyometrial route (12, 13, 29). Intravenous administration of carbetocin possesses a half-life of approximately 30–40 min, which is 4–10 times more sustained compared with that of oxytocin (9). When carbetocin was administered given via intramuscular injection, it achieves its highest plasma levels in under 30 min and demonstrates a rough bioavailability of 80% (9). Carbetocin demonstrates swift commencement of action regardless of drug route, yet its duration of action extends further after intramuscular injection. Rhythmic uterine contractions persist for 60 and 120 min after intravenous and intramuscular injections, respectively (9). Carbetocin is generally safe, and none of the meta-analyzed studies in our review reported major side effects (10–14).

Due to its abovementioned favorable pharmacokinetic and pharmacodynamic properties (8, 9), carbetocin can be beneficial in controlling uterine atony and excessive bleeding. High-quality evidence from meta-analysis reports highlighted the tolerability and efficacious superiority of carbetocin compared with oxytocin in preventing postpartum hemorrhage among women undergoing vaginal and cesarean section deliveries (6, 7, 30). Our current analysis expands the clinical applicability of perioperative carbetocin in myomectomy. The results of the current investigation indicate that carbetocin significantly reduces intraoperative blood loss, postoperative hemoglobin drop, and operation time. Moreover, carbetocin effectively lowers the need for perioperative blood transfusions. These findings hold particular significance in environments with limited resources, where access to blood bank facilities may be scarce. Additionally, given the scarcity of blood products and the potential risks associated with transfusions (31), the use of perioperative carbetocin may offer more valuable. However, from a clinical perspective, it is important to note that carbetocin is primarily indicated for the prevention of postpartum hemorrhage (6, 7, 30). Its use for preventing intraoperative bleeding during myomectomy is currently investigational and has not yet become a standard of care in clinical practice.

In the context of postpartum hemorrhage prevention, several cost-effectiveness studies from the United Kingdom (32, 33), Columbia (34), China (35), and India (36) have demonstrated the superiority of carbetocin over oxytocin (and misoprostol) in reducing major adverse maternal complications (such as death, hysterectomy, and postpartum bleeding >500 mL), lowering healthcare costs, and improving quality-adjusted life years (QALYs). However, for myomectomy, focused cost-effectiveness studies are needed to clarify the benefits of carbetocin compared to other hemostatic pharmacologic agents.

Our study possesses several notable strengths that warrant emphasis. To our knowledge, this is the first meta-analysis examining the tolerability and clinical utility of perioperative carbetocin compared to passive control interventions in abdominal myomectomy. By exclusively analyzing RCTs, we aimed to consolidate robust conclusions. We meticulously reported numerous efficacy endpoints to facilitate a thorough understanding of the findings. Furthermore, the robustness of our conclusions was reinforced by thorough analyses, including sensitivity analyses and certainty of evidence grading.

However, our study also presented some constraints that ought to be acknowledged. The limited quantity of RCTs included, along with their respective sample sizes, represents a significant constraint. Additionally, a few endpoints displayed heterogeneity, which may be attributed to alterations in perioperative considerations such as patient characteristics, clinical features of uterine leiomyomas, and drug routes. These considerations could affect the conclusions drawn from the pooled endpoints. As the number of included studies was small (*n* < 10), publication bias should be interpreted with caution (37). Additionally, since all studies originated from Egypt, there is a risk of publication bias and limited generalizability due to potential geographic, ethnic, and healthcare system differences (for example, availability of carbetocin). Future research should include more diverse populations across multiple countries to improve external validity. To the best of our knowledge, we were unable to find any observational studies in the literature comparing carbetocin to a placebo. Furthermore, some eligible RCTs exhibited some concerns regarding allocation concealment, potentially undermining the credibility of our conclusions.

Given the constraints, future research should focus on performing multicenter, large, and well-controlled RCTs comparing perioperative carbetocin use to passive control (placebo) during myomectomy. Prospective research could explore the varying efficacy of perioperative carbetocin based on different surgical techniques employed during myomectomy. Further inquiry may delve into the clinical effect of diverse routes of carbetocin (intravenous versus intramyometrial versus combination) in this patient population. Additionally, an intriguing avenue for future research would involve comparing carbetocin to active agents such as vasopressin, misoprostol, or tranexamic acid. Furthermore, future studies should investigate the potential additive efficacy of combining carbetocin with common active drugs to minimize blood loss during myomectomy.

## Conclusion

In patients undergoing abdominal myomectomy, the administration of prophylactic carbetocin during the perioperative period was generally tolerable and accompanied by noteworthy decreases in intraoperative bleeding, hemoglobin drops, blood transfusions, and operative time. Given the limitations of all studies originating in Egypt, small sample sizes, and the low to very low certainty of evidence, we emphasize that, while the pooled results suggest a potential benefit, the current evidence base is not sufficiently robust to support strong clinical recommendations. Further large-scale, multicenter, high-quality international RCTs are needed to validate these findings. Finally, the use of carbetocin for preventing intraoperative bleeding during abdominal myomectomy remains investigational and has not yet been established as a standard of care in clinical practice.

## Data Availability

The original contributions presented in the study are included in the article/Supplementary material, further inquiries can be directed to the corresponding author.
