# Supplementary material for: A meta-analysis of randomized controlled trials examining the effectiveness of carbetocin in reducing intraoperative blood loss during abdominal myomectomy
Source: Front Med (Lausanne). 2025 Jun 11;12:1590144. doi: 10.3389/fmed.2025.1590144 (PMC12187817; doi:10.3389/fmed.2025.1590144)
Supplement: Supplementary file 1 [file Table_1.docx]

**Supplementary Table 1**. The search strategy used in all databases.

| **PubMed**  All Fields: (carbetocin OR duratocin OR papal) AND (myomectomy) |
| --- |
| **Scopus**  TITLE-ABS-KEY ( ( carbetocin OR durabolin OR papal ) AND ( myomectomy ) ) |
| **Web of Science**  All Fields: (carbetocin OR duratocin OR papal) AND (myomectomy) |
| **Embase**  Broad search: ('carbetocin'/exp OR carbetocin OR 'duratocin'/exp OR duratocin OR papal) AND ('myomectomy'/exp OR myomectomy) |
| **Cochrane Central Register of Controlled Trials (CENTRAL)**  Title Abstract Keyword: (carbetocin OR duratocin OR papal) AND (myomectomy) |
| **Google Scholar**  All Fields: (carbetocin OR duratocin OR papal) AND (myomectomy) |
